# Eugenol-Loaded Nanocarriers Exert Particle-Specific Adverse Effects on Populations

**DOI:** 10.1021/acs.est.5c03624

**Published:** 2025-07-30

**Authors:** Bregje W. Brinkmann, Lan Dupuis, Sam Houdijk, Pelle Wattel, Cástor Salgado, Andrea Brunelli, José F. Fernández, Willie J.G.M. Peijnenburg, Martina G. Vijver

**Affiliations:** 1 Leiden University, Institute of Environmental Sciences (CML), Leiden 2333CC, The Netherlands; 2 Encapsulae SL, Castellón de la Plana 12006, Spain; 3 Department of Environmental Sciences, Informatics, and Statistics, 117711 Ca’ Foscari University of Venice, Venice 30172, Italy; 4 119906 Instituto de Ceramica y Vidrio, ICV-CSIC, Madrid 28049, Spain; 5 Center for Safety of Substances and Products, National Institute of Public Health and the Environment (RIVM), Bilthoven 3720BA, The Netherlands

**Keywords:** advanced materials, nanocarriers, crop protection products, environmental safety, automated video tracking

## Abstract

Nanocarriers provide promising prospects for the transition to more sustainable agrochemical practices. However, the unique release dynamics of their loaded chemicals raise concerns about potential adverse effects on nontarget organisms. To address this, we compared the toxicity of bentonite and sepiolite nanocarriers loaded with the anesthetic/antibacterial chemical eugenol to the toxicity of pure eugenol. was exposed to loaded nanocarriers, pure eugenol, and bare nanocarriers. In acute immobilization tests, a 50% effect concentration (EC_50_) of 0.14 ± 0.01 mg eugenol L^–1^ was derived for pure eugenol. Loading eugenol onto bentonite and sepiolite nanocarriers mitigated this acute toxicity, as indicated by respective EC_50_ values of 0.48 ± 0.02 and 0.57 ± 0.07 mg eugenol L^–1^. In 12-day toxicity tests, similar concentrations of eugenol were released from both nanocarriers. For the first day of exposure, this temporarily reduced the swimming speed of the daphnids. Moreover, in contrast to sepiolite nanocarriers, bentonite nanocarriers induced marked decreases in population growth. This reveals that nanocarriers exert particle-specific adverse effects on daphnid populations that cannot be predicted based on the toxicity of their individual constituents. We therefore plead to assess nontarget effects of the complete nanocarrier system, including the carrier and its loading, before allowing these products on the market.

## Graphical abstract

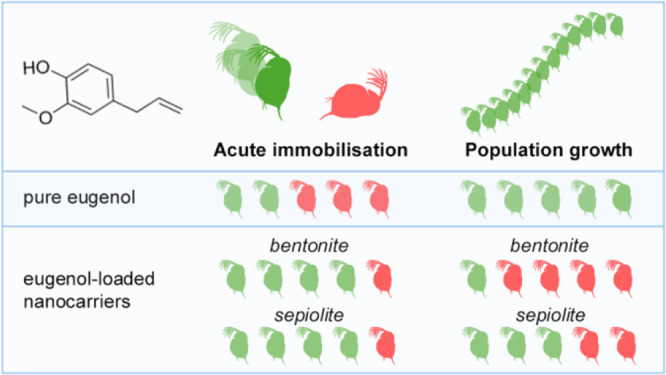

## Introduction

Advances in nanotechnology have led to the creation of nanocarrier systems to tackle modern challenges in medicine, agriculture, food, and energy supply.^1,2^ These systems, as defined by Gressler et al.,^3^ include “any material, material combination, chemical substance, compound, or structure with at least one dimension smaller than 1000 nm, capable of encapsulation or binding an active ingredient, aiming, among other things, to protect, disperse, transport, or sustain the release of the active ingredient and thereby enhancing its efficacy and/or safety”. For agricultural applications, nanocarriers complement other nanoenabled solutions in which the active ingredient (AI) is applied in a nanoform (e.g., metal oxide nanoparticles).^4^ Crucially, nanocarrier crop protection products aim for a targeted and sustained exposure over time, employing the known efficiency of conventional pesticides combined with the inherent properties of nanomaterials to modulate the release of the AI.^5–7^ This can be achieved through various designs, including the covalent binding or electrostatic attachment of the AI onto the surface of the carrier material, the adsorption of the AI into pores or in between branches of nanostructures like nanosponges or nanodendrimers, or by encapsulating the AI into capsules, such as nanoliposomes, empty pollens, virus capsids, or spore shells.^3^ Owing to their lower effective dose and lower required application frequency, these systems have been suggested to decrease the environmental burden of pesticides.^8^

Nanocarriers have been proven to be more efficient in managing pests than metal-based nanoformulations and conventional agrochemicals in both lab and field-based experiments.^4^ Additionally, a growing body of work indicates that pesticide-loaded nanocarriers may have equal or lower acute toxicity toward nontarget species as compared to conventional analogues.^9–11^ However, the very properties that make nanocarriers appealing also raise questions about their potential toxicity to the environment.^12–14^

Most toxicity studies for nanocarriers are performed based on standardized acute toxicity tests, which do not capture the dynamic fate that is known for the release of the AI from nanocarriers.^15–18^ Environmental conditions, including the pH, ionic strength, and presence of biomolecules, can influence the fate and behavior of nanocarrier systems.^19^ For example, high ionic strength and the formation of biomolecular coronae can enhance the agglomeration of nanocarriers, while low pH and reducing redox conditions can promote the degradation and release of the AI from nanocarriers. To be able to capture the effects of slowly released chemical loadings from newly developed nanocarrier systems, fate and toxicity data need to be integrated over relevant time spans and under realistic environmental conditions, as shaped by nontarget biota. Without such understanding, there is a risk that nanoenabled pesticides that are applied to agricultural fields and unintendedly enter surface waters will induce adverse effects on a wide range of aquatic species.^20–22^

In view of these considerations, we investigated how the toxicity of nanocarrier systems relates to the toxicity of the released AI, by characterizing the fate and toxicity of the released AI to the aquatic nontarget organism (D. magna). We focus on different nanoclay carriers loaded with a clove essential oil (CEO) extract, with the aim to determine (1) whether the long-term impacts induced by the nanocarriers can be extrapolated from their acute toxicity and (2) whether those effects are fully driven by the AI or depend on the nanocomponent. Nanoclays offer a versatile platform for the development of nanocarriers due to their biocompatibility, low toxicity, and cost-effectiveness.^23–25^ The main AI of the CEO loading is eugenol (C_10_H_12_O_2_; 4-allyl-2-methoxyphenol), which is commonly used in the food and feed industry and in fisheries as an additive and an anesthetic.^26,27^ Eugenol additionally exhibits wide range-of-action pesticidal and antibacterial properties.^28–30^ However, eugenol degrades quickly in the aquatic environment as a result of photooxidation.^31^ The clay-based nanocarriers can delay this rapid degradation through the retarded release of adsorbed eugenol, thereby enhancing and extending the duration of the potential adverse effects of eugenol on aquatic organisms.

In the present study, we compared the toxicity of two different nanocarriers to neonates and populations: a layered bentonite nanocarrier and a fibrous sepiolite nanocarrier, both of which were loaded with CEO. The crustacean was selected as the test species in view of its sensitivity to a large range of compounds, including the AI eugenol^32,33^ and bare nanocarriers.^34^ Automated tracking software was employed to track the survival, reproduction, and swimming speed of daphnids from populations that were exposed to eugenol in its pure form and to each of the nanocarrier systems. Quantification of actual concentrations of released eugenol in these tests revealed that both nanocarriers were applied at equivalent concentrations of released eugenol in the exposure medium. Owing to this equivalent dosing, the comparison between effects of both nanocarriers in the present study allows evaluation of how nanocarrier-specific characteristics can shape adverse effects on nontarget organisms.

## Materials and Methods

###  Cultures

 were reared as per OECD 211 recommendations.^35^ Stock cultures were maintained in Elendt M7 medium at 22 ± 1 °C with a 16 h:8 h (light:dark) photoperiod cycle. The rearing medium was oxygenated continuously, and its pH was maintained between 7 and 9. The cultures were fed *ad libitum* with live () three times a week. Neonates obtained from the culture were tested twice a year for their sensitivity to K_2_CrO_7_.^36^ The results of this reference test complied with ISO 6341.2012 (0.6 mg L^–1^ < EC_50,24h_ < 2.1 mg L^–1^).

### Preparation of Exposure Media

Liquid eugenol (CAS 97-53-0; purity ≥99%) was purchased from Sigma-Aldrich (Zwijndrecht, Netherlands). Bentonite and sepiolite nanocarriers were provided by SEPIOLSA (Minersa Group, Guadalajara, Spain) and consisted of layered and fibrillar nanoclay minerals. The nanocarriers were suspended in water media with 10% wt/wt of solids by a hand blender and stored for 24 h before stirring with a Cowles-type mixer (Dissolver SL-1 Heidolph RZR 2102) at 1200 rpm for 20 min and filtered with a 100 μm sieve. The slurries were transferred to a magnetic filter coupled to a Xylem Flojet pump in order to remove iron-based impurities. The resulting suspensions were delaminated or defibrillated by using an ultraturrax (IKA with rotor 8003300 T50) homogenizer, and the suspension was heated up to 60 °C. Both nanocarriers were loaded by the manufacturer with CEO. The CEO consisted of 16% wt/wt caryophyllene (CAS 87-44-6), 84% wt/wt eugenol, and 0.02% carvacrol. CEO was loaded into the nanocarrier at a final concentration of 19–20% wt/wt (for the bentonite nanocarrier) and 7–8% wt/wt (for the sepiolite nanocarrier). In addition, the bentonite nanocarrier was stabilized with 1–2 wt/wt % fumaric acid, and the sepiolite nanocarrier was coated with 3–4% wt/wt octadecylamine and stabilized with 2–3% wt/wt fumaric acid. The loaded nanocarriers were dried in a P-Selecta 2002972 DryBig oven at 80 °C for 48 h. Finally, the loaded nanocarriers were ground with an ultracentrifugal mill ZM300 (Retsch). In methanol extracts from both nanocarriers, 99.97% eugenol, only 0.03% caryophyllene, and no carvacrol were detected by gas chromatography coupled to mass spectrometry (GC-MS). Therefore, this study focused entirely on eugenol. Stock solutions and dispersions were prepared for (1) pure eugenol, (2) bare bentonite and sepiolite particles, (3) loaded bentonite and sepiolite nanocarriers, and (4) combined treatments, consisting of bare nanocarrier suspensions spiked with pure eugenol. From here on, the complete nanocarrier systems, including their loading, are simply referred to as “nanocarriers”, while the bare nanocarriers are specifically named “bare nanocarrier” or “bare bentonite/sepiolite particles”.

To prepare the eugenol stock solution, eugenol was first dissolved in 96% ethanol at 7.5 g L^–1^ (v. eq). Thereafter, the stock was diluted in Elendt M7 medium to reach a final concentration of 500 mg eugenol L^–1^. The solution was vigorously stirred for 10 min using a magnetic stirrer. Stock suspensions of both nanocarriers in their bare and loaded forms were prepared at a final concentration of 1 g L^–1^ in M7 medium. The suspensions were magnetically stirred for 60 min at 1300 rpm using a magnetic stirrer. For combined treatments, stock suspensions of bare bentonite and sepiolite nanocarriers were spiked with pure eugenol at eugenol concentrations of 19 (w/w) and 6.9% (w/w), respectively. These percentages correspond to the eugenol content in the loaded nanocarriers. Freshly prepared stocks were vortexed immediately prior to exposure and diluted to the exposure concentrations in M7 medium (Table 1).

**Table 1:** Comparison of Nominal and Actual Concentrations of Eugenol in Acute Toxicity Tests^a^

treatment	nominal [nanocarrier] (mg L^–1^)	nominal [eugenol] (mg L^–1^)	actual [eugenol] (mg L^–1^)	eugenol release rate (%)	AUC (mg L^–1^ 2d)
pure eugenol	0.0	0.0	<LOD	NA	NA
	0.0	0.1	0.16 ± 0.001	NA	0.10
	0.0	0.25	0.29 ± 0.002	NA	0.17
	0.0	0.5	0.54 ± 0.03	NA	0.37
	0.0	1.0	1.59 ± 0.01	NA	0.72
	0.0	2.0	3.19 ± 0.04	NA	1.37
	0.0	5.0	5.80 ± 0.06	NA	2.05
bentonite nanocarrier	0.0	0.0	<LOD	NA	NA
	10	1.9	0.39 ± 0.006	20.5	0.18
	25	4.8	0.98 ± 0.001	20.4	0.37
	50	9.5	1.85 ± 0.02	19.5	0.70
	100	19	3.22 ± 0.02	16.9	1.07
	200	38	6.40 ± 0.09	16.8	2.24
	500	95	15.90 ± 0.07	16.7	6.18
sepiolite nanocarrier	0.0	0.0	<LOD	NA	NA
	10	0.7	0.24 ± 0.02	34.2	0.17
	25	1.7	0.57 ± 0.003	33.5	0.30
	50	3.5	1.05 ± 0.01	30.0	0.50
	100	6.9	2.09 ± 0.008	30.3	0.89
	200	13.8	4.15 ± 0.09	30.1	1.63
	500	34.5	9.70 ± 0.05	28.1	3.10

a Abbreviations: CEO, clove essential oil; LOD, limit of detection; NA, not applicable. Total nanocarrier weights (nanocarrier and loading) are presented. Eugenol concentrations and release rates correspond to the initially released eugenol concentrations. The total area under curves (AUC) was derived from aqueous eugenol concentrations measured over the full exposure duration (Figure S6).

### Exposure Characterization

Exposure media were characterized in terms of (1) the primary particle size and shape of bare and loaded nanocarriers, (2) the zeta potential and hydrodynamic of aggregates from loaded nanocarriers, and (3) the released concentrations of eugenol, measured over the full exposure duration.

The primary particle size and shape were characterized by transmission electron microscopy (TEM). To this end, 10 μL of 10 mg L^–1^ bare and loaded nanocarrier suspensions was transferred to copper-mesh grids. The grids were allowed to dry for at least 24 h. Thereafter, particles were imaged using a JEOL 1400 microscope (JEOL Ltd., Japan) operating at 120 kV and a JEOL 2100F operating at 200 kV with a field emission gun.

The hydrodynamic size and zeta potential were determined for loaded nanocarrier suspensions of the medium test concentration (Table 2) by using a Zetasizer Ultra instrument (Malvern Panalytical, United Kingdom). For these measurements, the absorption value was set to 0.01, and the reflection index was set to 1.5 for the bentonite nanocarrier^37^ and to 1.52 for sepiolite nanocarrier.^38^ Bare and loaded nanocarrier suspension stability was in situ monitored by using a Turbiscan lab backscattering stability analyzer (Microtrac Formulaction, France).

**Table 2:** Comparison of Nominal and Actual Concentrations of Eugenol in Population-Level Tests^a^

treatment	exposure level	nominal [nanocarrier] (mg L^–1^)	nominal [eugenol] (mg L^–1^)	actual [eugenol] (mg L^–1^)	eugenol release rate (%)	AUC (mg L^–1^ 12d)
bare bentonite	medium	23.5	0	<LOD	NA	NA
bentonite nanocarrier	low	17.0	3.4	0.34 ± 0.02	10.0	1.0
	medium	33.0	6.6	0.76 ± 0.02	11.2	3.1
bare sepiolite	medium	48.3	0	<LOD	NA	NA
sepiolite nanocarrier	low	26.0	1.8	0.33 ± 0.01	18.3	1.1
	medium	51.9	3.6	0.78 ± 0.01	21.7	3.3
pure eugenol	low	0	0.06	0.05 ± 0.002	NA	0.2
	medium	0	0.12	0.12 ± 0.003	NA	0.4

a Abbreviations: CEO, clove essential oil; LOD, limit of detection; NA, not applicable. Total nanocarrier weights (nanocarrier + loading) are presented. Eugenol concentrations and release rates correspond to initially released eugenol concentrations. The total area under curves (AUC) was derived from the aqueous eugenol concentration measured over the full exposure duration (Figure 3). Results for combined treatments are presented in Table S5.

Aqueous concentrations of eugenol were measured for all exposures by ultraperformance liquid chromatography–inductively coupled plasma–mass spectrometry (UPLC-ICP-MS). The instruments and settings that were used for these measurements are provided in the Supporting Information (Tables S2, S3, and S4).

### Toxicity Tests

The toxicity of eugenol, and the bare and loaded nanocarriers, was tested by way of acute and long-term toxicity tests.

Acute tests were conducted following the standard acute immobilization guideline of OECD.^36^ Five biological replicates were used for all concentrations. Test concentrations were selected based on a range-finding test and were aimed to include the lowest effect concentration, the 100% effect concentration, and three intermediate concentrations. Concentrations for the bare nanocarriers were based on concentrations of the loaded nanocarriers, by accounting for the weight of the loaded AI. These nominal concentrations, ranging from 0 to 5 mg pure eugenol L^–1^, from 0 to 500 mg nanocarrier L^–1^, and from 0 to 640 mg bare nanocarrier L^–1^, are presented in comparison to actual concentrations of eugenol in the Results section (Table 1). Five neonates (<24 h old) were transferred from the main culture to 50 mL beakers filled with 20 mL of exposure medium. Following Annex 1 of the test guideline, the immobilization rate was assessed after 24 and 48 h of exposure as the fraction of neonates that did not swim within 15 s after gentle agitation of the test vessel. Daphnids were not fed, and the medium was not oxygenated during exposure. Dissolved oxygen and pH were monitored during the experiment. At each time point, 2 mL of the exposure medium was sampled and centrifuged at 15,400*g* for 5 min. An aliquot of the supernatant was transferred to a 1 mL tube and kept in the dark at 4 °C for quantification of aqueous eugenol concentrations.

Long-term tests for 12 days were performed to investigate sublethal effects of the nanocarriers on populations. The exposure concentrations for this test were derived from acute test results, selecting concentrations corresponding to 20% (low) and 40% (medium) of the acute EC_50_ value. In addition to the loaded nanocarriers, bare nanocarriers, pure eugenol, and a treatment consisting of bare nanocarriers spiked with pure eugenol at concentrations corresponding to the loaded product were included. All nominal exposure concentrations are presented in comparison to actual exposure concentrations in Table 2 and Table S5. At the start of each test, 10 daphnids of 10 days old were transferred to 500 mL beakers comprising the exposure media. These exposure media were not artificially oxygenated. Four replicates of each exposure treatment were included. From each beaker, 2 mL of exposure medium was sampled on a daily basis and immediately centrifuged for 5 min at 15,400*g*. The supernatant of these samples was stored at 4 °C until quantification of the actual concentrations of released eugenol. At the same time points, population growth and swimming speed were assessed. During the 12-day exposure, daphnids were fed daily with fresh *R. subcapitata* algae, providing 0.5 mL of 8.7 × 10^5^ algal cells mL^–1^ to each population. Both the pH and dissolved oxygen concentrations were monitored over the full duration of the experiment.

### Automated Daphnid Tracking

The count and swimming speed of daphnids were determined by way of automated tracking on an almost daily basis from 0 to 12 days postexposure, excluding weekend days (as specified in Table S1). The setup described by Bruijning et al.^39^ formed the inspiration and starting point for the tracking procedure. In this section, we describe what adjustments were made to the recording, image preprocessing, and tracking phase, in order to differentiate daphnids from aggregates of algae and nanocarriers. Detailed settings for each of the phases can be found in the Supporting Information (Table S1).

Recordings of daphnid populations were recorded in transparent Plexiglas cuvettes. This allowed daphnids to perform their natural up- and downward swimming motion and moreover resulted in the spatial separation of swimming daphnids and settling aggregates of algae and nanocarriers. The content of each exposure beaker was carefully poured into the cuvette, was placed in a black cardboard box, and illuminated from the right and left side (Figure S1). Next, aggregates of algae and nanocarriers were allowed to settle for 30 s, 2.5 min recordings were made, and the content of the cuvette was carefully poured back into the corresponding exposure beaker.

Image sequences were generated using FFMPEG (v. 5.0.3). The sequences were trimmed to 30 s of footage from the center of each movie and were cropped to the outlines of the cuvette using the FIJI distribution of ImageJ2 (v. 2.14.0).^40^ A median filter with a 2 pixel diameter was applied to reduce noise from small algae and nanocarrier aggregates.

Tracking was performed using the R package Trackdem (v. 0.7.2).^39^ This package identifies moving objects by subtracting motionless pixels (i.e., the “still background”) from each frame of the image sequence. At three steps, we applied filters to differentiate daphnids from moving aggregates of algae and particles. First, identified particles (i.e., algae, nanocarriers, and daphnids) were filtered based on their size and intensity using the *partIden* function. Next, a neural network was trained using the *testNN* function to filter any remaining false detections. We note that, given the aim to differentiate algae and nanocarrier aggregates from daphnids, the neural network was trained based on a reference image sequence comprising both adults and neonates of as well as intermediate levels of clay and algae. Thereafter, tracks were computed using the *trackParticles* function, and any tracks spanning less than half of the recording were excluded. This prevented tracking the same daphnid twice and moreover ensured that tracks from large aggregates of algae or nanocarriers that settled to the bottom of the cuvette were omitted. The corresponding R script, detailed tracking settings, and recorded parameters and are included in the Supporting Methods and Tables (Table S1).

The quality of the acquired tracking data was assessed based on plots of all detected particles and particle trajectories (see Figure S2 for example plots). This revealed that tracking results that were recorded on day 8 and day 10 (bare bentonite, bare sepiolite, and pure eugenol) needed to be omitted due to tracking issues. Additionally, incorrect tracks corresponding to settling nanocarrier aggregates or reflections of daphnids on the walls of the cuvette were manually removed from the data of day 0, 1, and 2 of the experiment.

### Daphnid Swimming Speed

The average swimming speed (*v̅*) was calculated for the 10 adult daphnids that started populations following

$$\mathrm{v̅}=\frac{\Delta s}{\frac{1}{FPS}\cdot \left(f_{end}-f_{start}\right)}$$ 1

where Δ*s* is the sum of displacement between all consecutive frames of a track, FPS is the frame rate, and *f*_start_ and *f*_end_ are the numbers of the first and final frame of a track, respectively. Neonates were excluded from swimming speed analysis because these swim faster than adults and have been exposed to different concentrations of eugenol, depending on their time of hatching. A size threshold of 50 pixels (8.8 mm) was applied to achieve this.

### Data Analyses

Data analysis and visualization were performed in R (v. 4.4.1; www.r-project.org) using the packages “car” (3.1–3), “drc” (v. 3.0.1),^41^ “dplyr” (1.1.4), “lme4” (v. 1.1–35.5), “plotrix” (v. 3.8–4), “readr” (v. 2.1.5), and “stats” (v. 4.4.1). The mean and standard error of the mean (SEM) are reported.

Concentration–response curves, decay curves, and growth curves were fitted using the *drm* function. The four-parameter log–logistic model was used to obtain concentration–response curves, the four-parameter Weibull model was used to obtain decay curves, and the four-parameter logistic model was used to obtain growth curves. EC_50_ concentrations and slopes were compared by using *F*-tests of the drc-integrated *compParm* function. The area under the curve (AUC) was computed by using the *integrate* function.

The hydrodynamic size and zeta potential of aggregates from bentonite and sepiolite nanocarriers were compared using a Welch two-sample *t-*test. Replicate measurements were averaged prior to this comparison. The Shapiro–Wilk test for normality was applied to check if the sizes and zeta potentials of both nanocarriers followed a normal distribution. Additionally, Levene’s test was used to check if the variance of size and zeta potential measurements was equally spread across the bentonite and sepiolite nanocarrier types.

The final population size was assessed by comparing the total count of daphnids between each of the treatments, as listed in Table 2 and Table S5, on the final day of exposure using the one-way ANOVA test combined with Tukey’s HSD post hoc test. Five populations that collapsed following exposure to the bentonite nanocarrier and eight populations that collapsed following exposure to the combined treatment at medium effect levels were excluded from the analysis. The normality of model residuals was assessed by inspecting diagnostic plots and using the Shapiro–Wilk test for normality. Additionally, the equal spread of variance across all treatments was verified using Levene’s test.

The relationship between the swimming speed of adult daphnids and the concentrations of aqueous eugenol measured at the start of exposure (Table 2 and Table S5) was tested in a mixed-ANCOVA design using the *lmer* function. To account for the repeated measurement of populations from the same beaker, the beaker was included as the random effect. Results for exposure day 0, day 1, and day 2 were tested separately. Three tests were performed for each of these days. The first test included all populations that were exposed to the bentonite nanocarrier, pure eugenol, and the control. The second test included all populations that were exposed to the sepiolite nanocarrier, pure eugenol, and the control. The third test included all populations that were exposed to bare nanocarriers, either with or without the addition of pure eugenol. In the latter test, the clay type of the bare nanocarriers (bentonite or sepiolite) was included as a second explanatory variable, omitting the insignificant interaction between the clay type and aqueous eugenol concentration (i.e., “speed ∼ [aqueous eugenol] + clay type + (1|beaker)”). For all tests, the normality of model residuals was checked by inspecting histograms and QQ plots, generated using the *qqnorm* function. Additionally, residuals were plotted against fitted values, to check if model residuals distributed equally across each of the compared eugenol concentrations. Finally, *p*-values were computed based on a type III analysis of variance with Satterthwaite’s method, using the *anova* function.

## Results

### Nanocarrier Size and Shape

The primary particle size and shape of the bentonite and sepiolite nanocarriers in their bare and loaded form were investigated by way of TEM microscopy (Figure 1 and Figure S3). The size of aggregates of (loaded) bentonite and sepiolite nanocarriers was additionally studied by dynamic light scattering (Figure S4).

**Figure 1:**
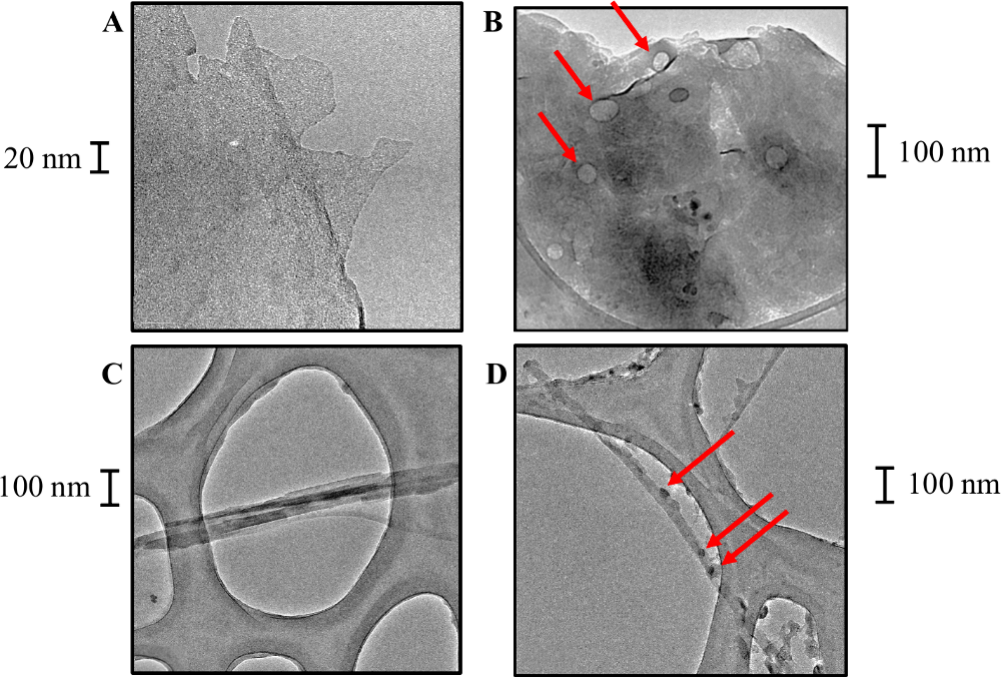
Transmission electron microscope micrographs of bare bentonite particles (A), the bentonite nanocarrier (B), bare sepiolite particles (C), and the sepiolite nanocarrier (D). Red arrows indicate CEO nanodrops in the loaded nanocarriers.

Bare bentonite particles and bentonite nanocarriers were flake-shaped and had a primary size averaging 142.5 ± 3.4 nm and a length-to-width ratio of 1.6 ± 0.1 (*n* = 10; Figure 1A,B). Due to the low electron density of these layered type particles, individual bentonite particles were hardly discernible when aggregated. Smaller particles (10–35 nm diameter) with higher electron density could be observed in the form of dark aggregates (Figure S3A) and correspond to amorphous silica particles in the form of opal nanoparticles (an amorphous form of silica, SiO_2_·*n*H_2_O). Opal nanoparticles can form naturally in sedimentary environments, where silica-rich solutions precipitate under low temperatures. The similarity of this contamination for the bare bentonite particles and bentonite nanocarriers still allows us to assess the effects of the bentonite nanocarrier based on the comparison of both materials. No differences in the primary particle size and shape could be observed between bare bentonite particles (Figure 1A and Figure S3A) and bentonite nanocarriers (Figure 1B and Figure S3B). In the exposure medium, the bentonite nanocarrier layered particles formed large aggregates with a hydrodynamic size and zeta potential averaging 2.9 ± 0.2 μm and −15.2 ± 0.8 mV, respectively, according to DLS measurements, Figure S4. Although the primary particles of clays are laminar or fibrillar, their agglomerates do present a greater sphericity, and the DLS measurements that correspond to agglomerates can be considered as an estimate of the size of the said agglomerates. The aggregation of nanocarriers was enhanced by the presence of fumaric acid encapsulant.

Bare sepiolite particles and sepiolite nanocarriers were needle-like shaped, with a length-to-width ratio of 23.5 ± 0.4 (*n* = 50; Figure S3C,D). The lengths of individual particles were highly heterogeneous and ranged from 20 nm to 5 μm. In contrast to the bentonite nanocarrier, essential oil was clearly visible as droplets on and around the needles of the sepiolite nanocarrier (Figure 1D). While the zeta potential of the sepiolite nanocarrier was significantly lower than that of the bentonite nanocarrier, averaging −20.7 ± 0.9 mV (*t* = 4.6, *df* = 4.0, and *p* = 0.01), sepiolite suspensions present larger aggregates than bentonite suspensions, with an average hydrodynamic size of 12.9 ± 5.2 μm, Figure S4. Due to the large spread in aggregate sizes, this mean size was not significantly different from the mean aggregate size for the bentonite nanocarrier (*t* = −1.9, *df* = 2.0, and *p* > 0.05). We furthermore note that we did not observe any peaks in the size distributions of both nanocarriers that correspond to the monolayer or solvation layer thickness of these materials, indicating that these remained unexfoliated and aggregated in the aqueous medium. While additional measurements of the sheet thickness (e.g., by way of atomic force microscopy) are required to confirm this, these observations are in line with the TEM images (Figure S3), exclusively showing multilayered sheets of bentonite flakes or sepiolite needles.

Regarding potential sedimentation dynamics, we did not observe significant differences in the mean hydrodynamic size of both the bentonite and the sepiolite nanocarrier following 1 and 2 days of exposure (Figure S4A,B). However, we observed a clear decrease in the derived count rate following 1 day of exposure for the bentonite nanocarrier (Figure S4C) and following 2 days of exposure for the sepiolite nanocarrier (Figure S4D).

On the contrary, in situ suspension stability studies (Figure S5) demonstrated that the higher hydrodynamic size of sepiolite bare and sepiolite nanocarriers resulted in higher sedimentation than bentonite bare and bentonite nanocarrier suspensions. This sedimentation behavior is in agreement with the lower values of zeta potential. However, it is worth noting that in both cases, a lower transmittance was obtained for the loaded nanocarriers against their bare counterparts as an indicator of the presence of delaminated and defibrillated particles in the medium. This indicates that, while the bentonite nanocarrier appears to be somewhat more stable than the sepiolite nanocarrier, both nanocarriers settled over time. This indicates that, while the sepiolite nanocarrier appears to be somewhat more stable than the bentonite nanocarrier, both nanocarriers settled over time.

### Acute Toxicity

The acute toxicity of bentonite and sepiolite nanocarriers was compared by way of the *Daphnia* acute immobilization test.^36^ Nominal and actual exposure concentrations of eugenol in acute toxicity tests are compared in Table 1. While exposure to both the sepiolite nanocarrier and pure eugenol resulted in a maximal immobilization rate of 100% (Figure 2), a maximum immobilization rate of only 83.6 ± 6.9% was reached following exposure to the bentonite nanocarrier. These results indicate that the toxicity of the sepiolite nanocarrier was higher at high exposure concentrations than the toxicity of the bentonite nanocarrier. However, based on nominal nanocarrier concentrations (Figure 2A), a lower median effect concentration (EC_50_) was obtained for the bentonite nanocarrier (83.6 ± 6.9 mg nanocarrier L^–1^) than for the sepiolite nanocarrier (130 ± 16.6 mg nanocarrier L^–1^; *t* = 2.4, *p* = 0.02). To investigate if this difference can be explained by the release of eugenol (the main AI of the loaded CEO), the toxicity of bentonite and sepiolite nanocarriers was next compared based on actual concentrations of released eugenol. Based on these actual concentrations of eugenol (Figure 2B), similar median effect levels were derived for the bentonite nanocarrier (0.48 ± 0.02 mg eugenol L^–1^) and the sepiolite nanocarrier (0.57 ± 0.07 mg eugenol L^–1^). The median effect level for pure eugenol was lower, averaging 0.14 ± 0.01 mg eugenol L^–1^ (*t* = 8.2 and *t* = −20.6 as compared to bentonite and sepiolite nanocarriers, respectively; *p* < 0.001). No immobilization was observed for daphnids that had been exposed to bare nanocarriers at concentrations corresponding to the highest test concentrations of the loaded nanocarriers (600 mg of bare nanocarrier L^–1^). Combined, these results suggest that the acute toxicity of both nanocarriers is primarily driven by the release of eugenol into the medium.

**Figure 2:**
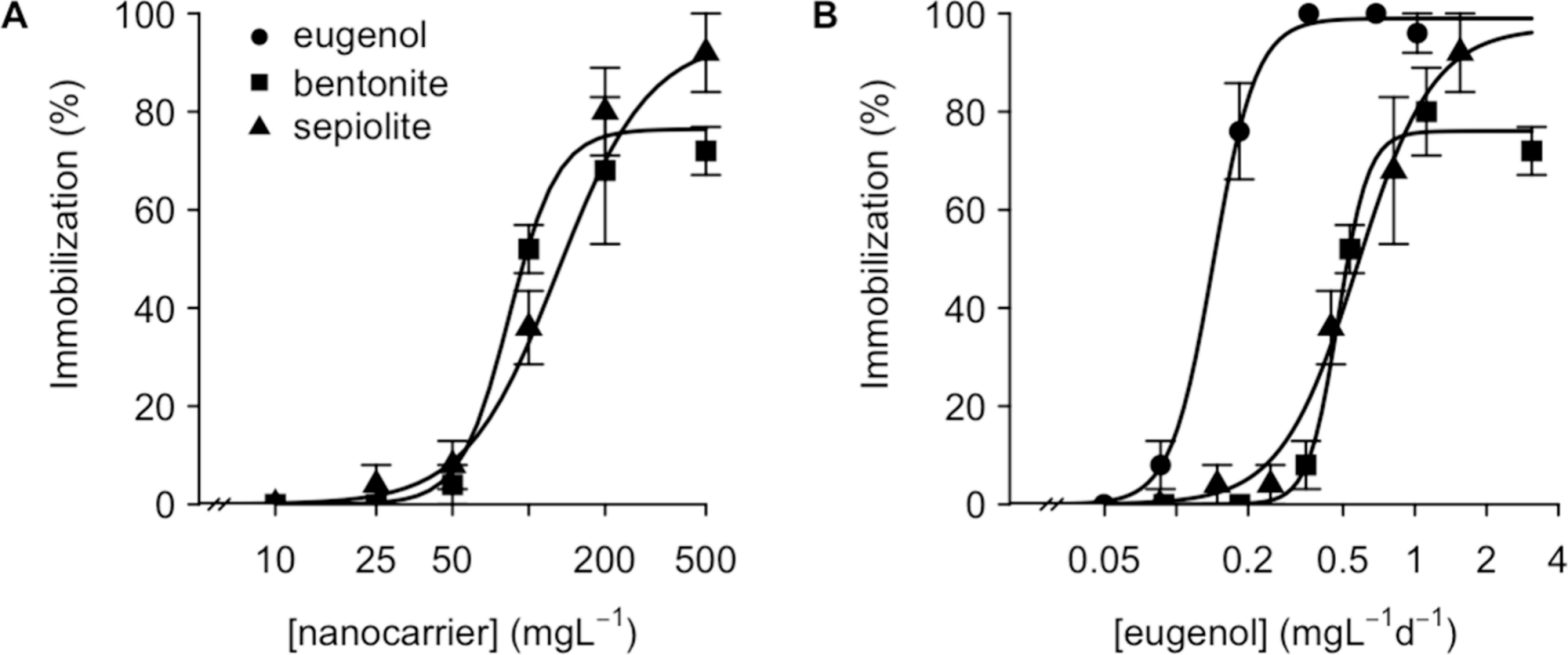
Fraction of immobilized neonates following 2 days of exposure to eugenol (circles), the bentonite nanocarrier (squares), and the sepiolite nanocarrier (triangles). Panel A presents concentration–response curves based on nominal nanocarrier concentrations. Panel B presents concentration–response curves based on the average actual concentrations of released eugenol per exposure day.

### Population-Level Exposure Dynamics

Based on the acute toxicity test, we subsequently investigated the effects of bentonite and sepiolite nanocarriers on *D. magna* populations at 20 and 40% of the acute toxicity EC_50_ values (Table 2).

Immediately following dispersion in Elendt M7, both nanocarriers released ∼10–20% of their eugenol loading into the exposure medium (Table 2). The initial concentrations of eugenol did not differ between bentonite (Figure 3A) and sepiolite nanocarriers (Figure 3B). However, aqueous eugenol dissipated from the exposure medium in a nanocarrier-specific manner. For the bentonite nanocarrier, aqueous eugenol concentrations decreased gradually from the start of the experiment onward, similar to the dissipation of eugenol without the addition of (bare) nanocarriers (Figure 3C and Table 2). In contrast, aqueous concentrations of eugenol remained stable in treatments with the sepiolite nanocarrier for the first 2 days of exposure, which was followed by a steeper decline of eugenol concentrations in the exposure medium. This behavior is in agreement with the higher sedimentation of the sepiolite nanocarrier and the low water solubility of the fumaric acid (0.63 g/100 mL) as the encapsulant. The low solubility of the encapsulant constrained the release of CEO nanodrops, and this process is in turn impeded by the presence of aggregates. Thus, the larger hydrodynamic radius of the sepiolite aggregates acts as containers that release the CEO more gradually for sepiolite than for bentonite nanocarriers. Nevertheless, the total aqueous exposure to eugenol over 12 days of exposure, calculated as the area under the curve (AUC), was similar for both nanocarriers (Table 2). The concentration of eugenol in negative controls and bare nanocarrier treatments remained below the detection limit over the full duration of the experiment, confirming that these treatments were free of eugenol contamination.

**Figure 3:**
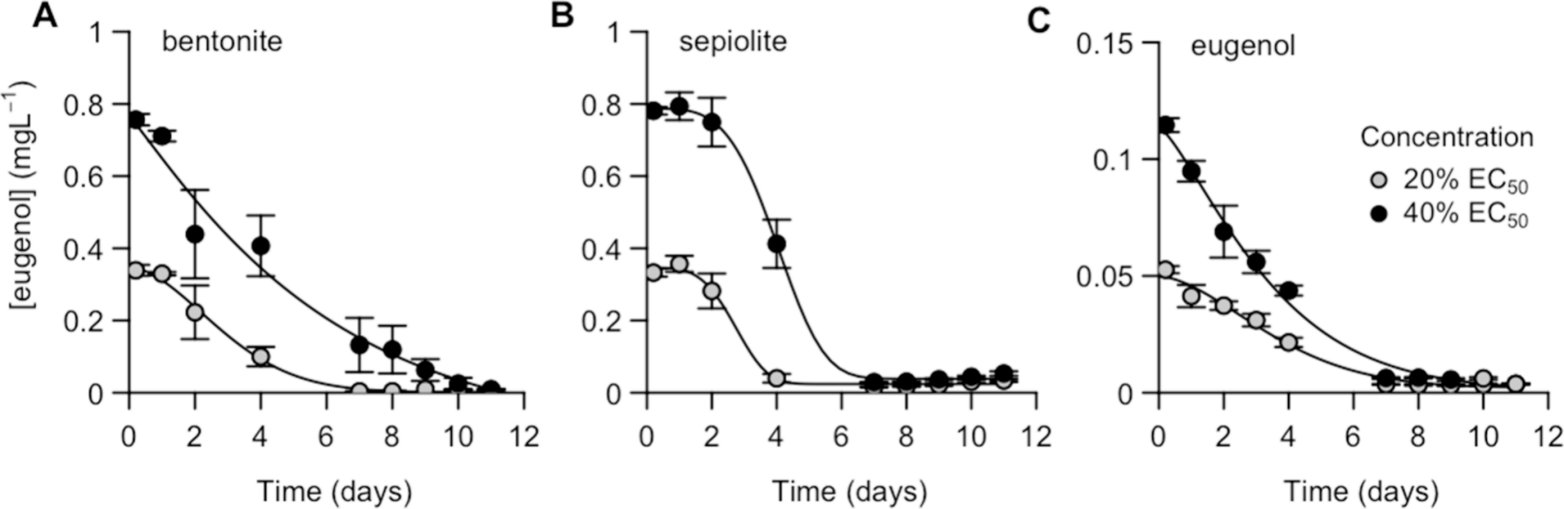
Aqueous eugenol concentrations in population-level tests for the bentonite nanocarrier (A), the sepiolite nanocarrier (B), and pure eugenol (C). Means and SEM are presented (*n* = 4) for the low exposure concentrations (gray circles: 20% acute EC_50_ values) and medium exposure concentrations (black circles: 40% acute EC_50_ values). Decay curves were fitted based on the four-parameter Weibull model. Results for combined treatments of bare nanocarriers and eugenol are presented in Figure S6.

When eugenol was mixed with the bare bentonite nanocarrier (Figure S6A) or the bare sepiolite nanocarrier (Figure S6C) at (nominal) concentrations corresponding to the loading of both nanocarriers, this resulted in much higher concentrations of eugenol in the exposure medium (Table S5). This fact confirms that the encapsulant plays an important role in the slow release of CEO from the nanocarriers. As previously mentioned, the low solubility of fumaric acid slows the release of the nanodroplets, causing a prolonged effect of CEO in the medium. Conversely, if the CEO is only absorbed on the surface of the materials in the mixtures, then the release occurs very rapidly due to its hydrophobic nature. The measured concentrations of eugenol were consistently lower than the nominally applied concentrations of eugenol for these treatments (Table S6), despite high resemblance of measured and nominal concentrations in pure eugenol treatments without bare particles (Table 2). Based on the assumption that the difference between measured and nominal concentrations of eugenol resulted from the adsorption of eugenol to bare particles, corresponding adsorption rates of 3.69 ± 0.13% (w/w) and 1.10 ± 0.12% (w/w) can be calculated for the bare bentonite and bare sepiolite nanocarriers, respectively. However, the high concentrations of aqueous eugenol in mixed treatments, compared to the concentrations of released eugenol from nanocarriers, complicate a further comparison of the effects of these treatments. Therefore, results for the combined treatment are included in the Supporting Information.

### Population-Level Effects

Immediately following exposure, treatments with both bentonite and sepiolite nanocarriers resulted in lower daphnid swimming speeds, proportionally to the released concentrations of eugenol (*t* = −3.0, *df* = 25.1, and *p* = 0.005 for bentonite; *t* = −3.6, *df* = 19.3, and *p* = 0.002 for sepiolite; Figure 4A). Mixed treatments consisting of bare particles and eugenol also resulted in slightly lower swimming speeds (*t* = −3.8, *df* = 19.7, and *p* = 0.001), irrespective of the nanocarrier type (*t* = 0.22, *df* = 20.0, and *p* > 0.05; Figure S8A). Except for loaded bentonite nanocarriers, these effects dissipated over the next 2 days of exposure (Figure 4B,C and Figure S8B,C). In view of the dissipation of eugenol from the exposure medium at later time points and the lethal effects of the bentonite nanocarrier at these time points, we did not analyze swimming speed from 3 days of exposure onward.

**Figure 4:**
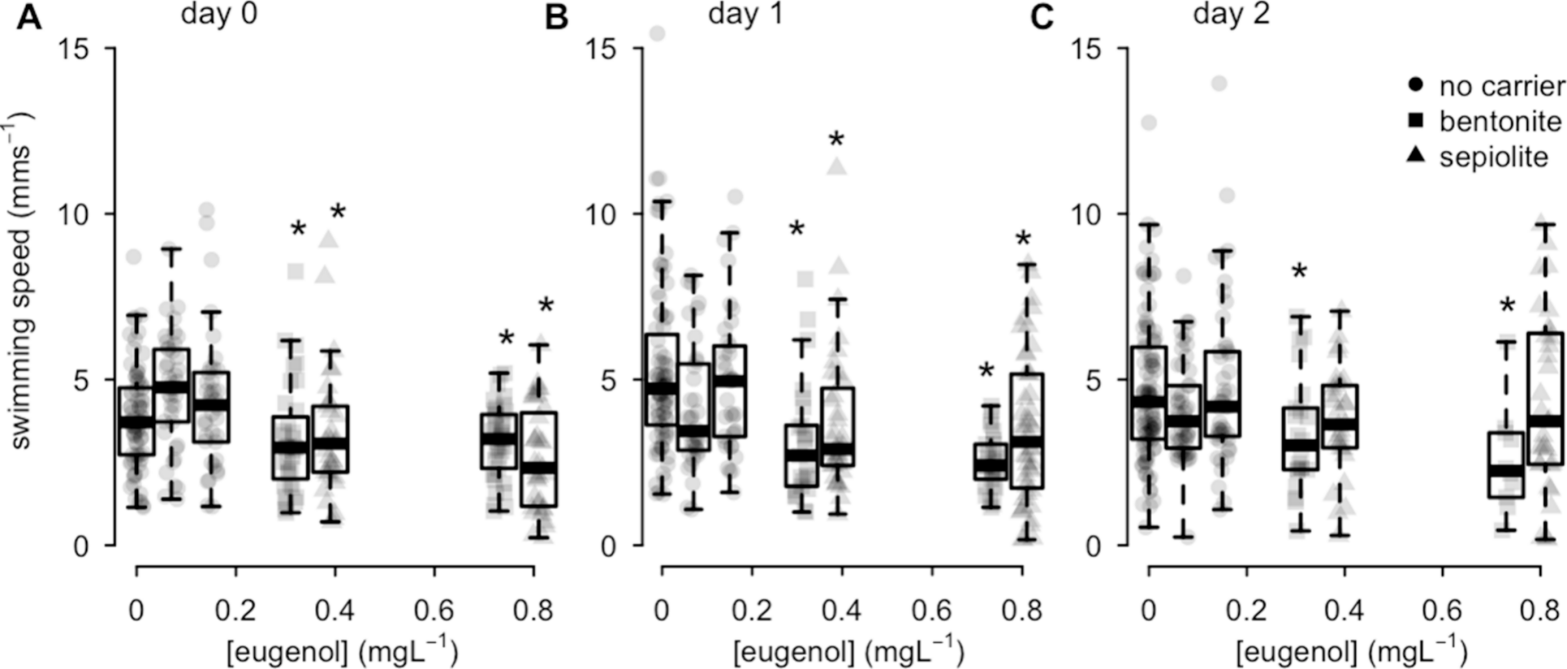
Swimming speed of individuals immediately following exposure (A), 1 day following exposure (B), or 2 days following exposure (C) to the bentonite nanocarrier (squares), the sepiolite nanocarrier (triangles), or pure eugenol (circles), as a function of the concentration of released eugenol. Results for bare particles, applied with or without the addition of eugenol, are presented in Figure S6. Significant relationships between the released eugenol concentrations and swimming speed (*p* < 0.05) are indicated with an asterisk above the boxplots of the nanocarriers.

Compared to the effects of the nanocarriers on the swimming speed (Figure 4), much more profound effects were observed on the growth of daphnid populations over the 12-day exposure time in terms of the daphnid count (Figure 5).

**Figure 5:**
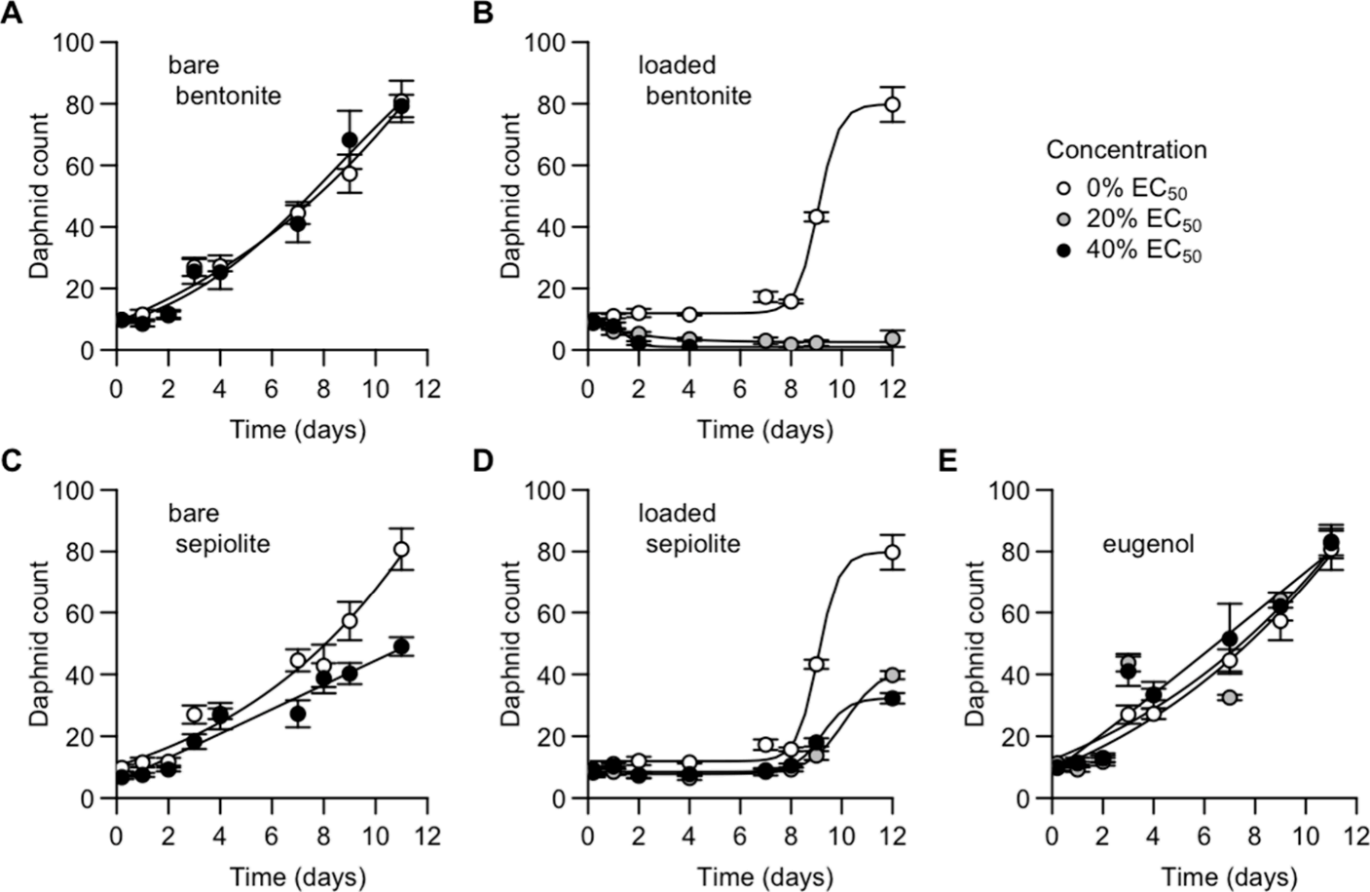
Growth of populations exposed to bare bentonite particles (A), the bentonite nanocarrier (B), bare sepiolite particles (C), the sepiolite nanocarrier (D), and pure eugenol (E). Means and SEM are presented (*n* = 4) for the low exposure concentrations (gray circles: 20% acute EC_50_ values) and medium exposure concentrations (black circles: 40% acute EC_50_ values). Growth curves were fitted based on the four-parameter logistic model.

All populations started with an initial population count of 10 daphnids per exposure beaker. In the absence of nanocarriers and eugenol, these populations increased in 11 to 12 days to a final count of 80 ± 4 individuals. Daphnid populations that were exposed to bare bentonite particles increased to a similar size of 79 ± 4 individuals at 11 days of exposure (Figure 5A). Remarkably, exposure to the bare sepiolite particles resulted in smaller population sizes, counting 49 ± 3 individuals at 11 days of exposure (*p* < 0.0001; Figure 5D).

When daphnids were exposed to the bentonite nanocarrier (Figure 5B) or applied to a mixture of bare bentonite particles and pure eugenol (Figure S7B), only 3 to 7 individuals survived to the end of exposure at the low exposure concentration. At the medium concentration, exposure to the bentonite nanocarrier even resulted in a full collapse of daphnid populations within 7 days. In contrast, when the sepiolite nanocarrier was applied (Figure 5D), daphnid populations still increased in size, albeit to lower population sizes than controls, counting 40 ± 1 and 32 ± 2 individuals at the low and medium exposure, respectively (Figure 5E; *p* < 0.001).

The effects of the mixed exposure to the bare sepiolite particles and eugenol on daphnid population growth were proportional to the added eugenol concentrations. Daphnid populations that were exposed to the low concentration of this mixture still reached a final population size of 38 ± 5 individuals at day 12, while populations that were exposed to the medium exposure fully collapsed within 7 days (Figure S7D). Exposures to pure eugenol did not impair population growth, irrespective of the exposure concentration, as assessed based on their final daphnid count, counting 83 ± 4 and 83 ± 5 individuals, respectively (Figure 5E).

## Discussion

A key aim for the development of nanocarrier systems for agricultural applications is to modulate the release patterns of adsorbed or encapsulated chemical loadings to obtain more sustained and controlled exposure patterns. This nanoenabled tuning of exposure patterns requires a thorough assessment of chemical fate and toxicity toward unintendedly exposed nontarget organisms.^18^ The regulation of nanocarrier systems that are applied as crop protection products in Europe (EC No. 1107/2009) is based on the assessment of the individual nanocarrier components (i.e., the active ingredient and the bare nanocarrier), where the active ingredient is additionally included in the REACH database of registered substances (EC No. 1907/2006, article 15). However, our present study focusing on clay-based nanocarriers loaded with eugenol demonstrates that the longer-term effects of nanocarriers do not necessarily follow the fate and effects of the released chemical loading and bare nanocarrier. As we discuss below, the prediction of longer-term adverse effects of nanocarriers therefore requires a more thorough understanding of the behavior of nanocarriers in their environment.

In line with previous studies,^42–44^ our acute toxicity tests indicate that nanoencapsulation mitigates the toxicity of the AI. Irrespective of the nanocarrier type, we observed a 3- to 4-fold decrease in the acute toxicity of eugenol upon loading. More specifically, we obtained 48 h EC_50_ levels of 0.14 ± 0.01 mg eugenol L^–1^ for pure eugenol, 0.57 ± 0.07 mg eugenol L^–1^ for the sepiolite nanocarrier, and 0.48 ± 0.02 mg eugenol L^–1^ for the bentonite nanocarrier. These values are in the same order of magnitude of previously reported EC_50_ levels,^45–47^ ranging from 0.7 to 2 mg eugenol L^–1^.

In marked contrast to the acute toxicity tests, the population-level experiments showed profound differences between the toxicity of bentonite and sepiolite nanocarriers. At equivalent concentrations of released eugenol, the bentonite nanocarrier impaired population growth much more than did the sepiolite nanocarrier. Similarly, while both nanocarriers initially impaired the swimming speed of , only daphnids that were exposed to the sepiolite nanocarrier recovered their swimming speed. Since no effects of bare bentonite nanocarriers could be observed, the higher toxicity of bentonite nanocarriers can only be attributed to interactions between the nanocarrier, its loading, and the exposed *D. magna.*

An important difference between the acute and population-level toxicity tests is the age of the daphnids that were used for these tests. Acute tests were executed with neonates, while population-level tests were executed with 10-day-old individuals who were fed. can ingest bacteria and algae of 0.6 μm up to 40 μm in size,^48^ although their ability to ingest “hard” materials is more restricted. The largest ingestible polystyrene bead for daphnids was found to be 2–5 times smaller than the largest ingestible alga.^49^ With respect to the nanocarriers, this could imply that daphnids can better ingest the smaller aggregates of the bentonite nanocarrier than those of the sepiolite nanocarrier. Once ingested, nanocarriers are exposed to the different environmental conditions of the gut lumen, such as the low pH, high ionic strength, and presence of biomolecules,^50^ which might affect the aggregation, residence time, and degradation of the nanocarrier,^51^ and can thereby influence the release of the AI. Therefore, the higher toxicity of the bentonite nanocarrier to daphnid populations could have resulted from the higher bioavailability of eugenol following its release from ingested nanocarriers.

Earlier, Vijver et al.^15^ proposed to include the time-dependent fate of nanomaterials in the ecotoxicological evaluation of nanoenabled products. Our results reveal that this is particularly relevant for nanocarriers, as acute toxicity tests did not allow an accurate assessment of toxicity caused by the dynamic interactions between the bare nanocarrier, its loading, and biota. This implies that the hazard assessment for nanocarrier crop protection products must be conducted on a case-by-case basis, that is, for the loaded product rather than its individual constituents and should include an evaluation of long-term effects. Additional *in vitro* tests should explicitly assess the influence of different environmental matrices, e.g., resembling the gut lumen of nontarget organisms,^51^ on the stability (OECD Test No. 318),^52^ degradation (OECD Test No. 301),^53^ and resulting release kinetics of the AI from nanocarriers.^54^ Moreover, adjustments to dispersion protocols should be made to prevent preliminary degradation of nanocarriers by, e.g., sonication procedures, resulting in the release of the AI prior to exposure. The first studies that investigate the toxicity of nanocarriers thoroughly evaluated size as the particle-specific property.^55,56^ Future directions should additionally include a detailed investigation of adsorption and desorption of the AI and assessment of the impacts of interactions between nanocarriers and their environment on the fate and toxicity of nanoenabled products over time. This is necessary to fully assess their potential to replace conventional agrochemicals and should therefore be included into routine testing prior to large-scale use.

## Supplementary Material



## Data Availability

Video footage and the trained neural network will be made publicly available via Zenodo once the manuscript has been accepted for publication. The trained neural network (R environment file (.RData) including the trained neural network for daphnid tracking analysis) and videos (image sequences) are available on Zenodo. DOI: 10.5281/zenodo.16165098.

## Notes

Details on UPLC and tracking methods, figures and tables for the combined treatments with bare nanocarriers and pure eugenol, and additional DLS measurements (PDF)
