# Correction to “Eugenol-Loaded Nanocarriers Exert Particle-Specific Adverse Effects on *Daphnia magna* Populations”

**DOI:** 10.1021/acs.est.5c11991

**Published:** 2025-09-11

**Authors:** Bregje W. Brinkmann, Lan Dupuis, Sam Houdijk, Pelle Wattel, Cástor Salgado, Andrea Brunelli, José F. Fernández, Willie J. G. M. Peijnenburg, Martina G. Vijver

In Exposure Characterization (page 16295), aqueous concentrations of eugenol were measured for all exposures by ultraperformance liquid chromatography-mass spectrometry (UPLC-MS). The analytical techniques did not include inductively coupled plasma (ICP).

In Nanocarrier Size and Shape (page 16298), the sepiolite nanocarrier appears to be somewhat more stable than the bentonite nanocarrier based on dynamic light scattering count rates (Figure S4C,D). The bentonite materials appear to be somewhat more stable than the sepiolite materials based on in situ suspension stability measurements (Figure S5).

References to the Supporting Information need to be corrected for results of the combined treatment of pure eugenol and bare particles (Table 1).

**Table 1:** Corrections to References to the Supplementary Material

result	section	incorrect reference	correct reference
aqueous concentrations of eugenol and population sizes	Population-Level Exposure Dynamics (page 16298), Figure 3 (page 16299)	Figure S6	Figure S7
swimming speed of *D. magna* individuals	Figure 4 (page 16299)	Figure S6	Figure S8
comparison of actual and nominal concentrations of eugenol	Population-Level Exposure Dynamics (page 16300)	Table S6	Table S5

An addition to the Acknowledgments has been made.

