# Supplementary material for: Eugenol-Loaded Nanocarriers Exert Particle-Specific Adverse Effects on Populations
Source: Environ Sci Technol. 2025 Jul 30;59(31):16293–303. doi: 10.1021/acs.est.5c03624 (PMC12355951; doi:10.1021/acs.est.5c03624)
Supplement: Supplementary file 1 [file es5c03624_si_001.pdf]

Supporting Information to:

Eugenol-Loaded Nanocarriers Exert Particle-Specific Adverse Effects to *Daphnia magna* Populations

Bregje W. Brinkmann[1‡], Lan Dupuis[1‡], Sam Houdijk[1], Pelle Wattel[1], Cástor Salgado[2], Andrea Brunelli[3], José F. Fernández[4], Willie J.G.M. Peijnenburg[1,5,], Martina G. Vijver[1]*

[1] Leiden University, Institute of Environmental Sciences (CML), Leiden, 2333CC, The Netherlands

[2] Encapsulae SL, Castellón de la Plana, 12006, Spain

[3] Department of Environmental Sciences, Informatics and Statistics, Ca' Foscari University of Venice, 30172, Italy

[4] Instituto de Ceramica y Vidrio, ICV-CSIC, Madrid, 28049 Spain

[5] National Institute of Public Health and the Environment (RIVM), Center for Safety of Substances and Products, Bilthoven, 3720BA, The Netherlands

‡ These authors contributed equally

Summary: 39 pages, 5 figures, 2 tables

CONTENTS

Supporting Methods____ page

R script for daphnid tracking …..…………………………………………………..……S2

UPLC protocol……………………………………………………………………..……S4

Supporting Tables____page

Table S1……………………………………………………………………………..……S6

Table S2……………………………………………………………………………..……S7

Table S3……………………………………………………………………………..……S7

Table S4……………………………………………………………………………..……S7

Table S5……………………………………………………………………………..……S8

Supporting Figures____page

Figure S1……………………………………………………………………….…….S9

Figure S2……………………………………………………………………….…….S9

Figure S3……………………………………………………………………….…….S10

Figure S4……………………………………………………………………….…….S11

Figure S5……………………………………………………………………….……S12

Figure S6……………………………………………………………………….……S12

Figure S7……………………………………………………………………….……S13

Figure S8……………………………………………………………………….……S13

SUPPORTING METHODS

R script for daphnid tracking

```
## 1. Libraries and neural network
library(trackdem)
library(readr)
load("NeuralNetwork.RData")

## 2. Footage settings
folders <- "./Fotosequences"
dirs <- list.dirs(folders, full.names = TRUE)[-1]
samples <- list.dirs(folders, full.names = FALSE)[-1]
fps <- 2 #footage frame rate
minFrames <- 31 #track length treshold

## 3. Creating a dataframe for tracking results
results <- as.data.frame(matrix(nrow = 0, ncol = 7))
colnames(results) <- c('Sample','ID','first_frame','last_frame',
                                         'detections', 'size', 'displacement')

## 4. Tracking
for (i in 1:length(dirs)) {
        print(i)
        print(samples[i])

        #4.1 image loading and pre-processing
        direc <- dirs[i]
        allFullImages <- loadImages(dirPictures = direc, nImages = 1:60)
        stillBack <- createBackground(allFullImages, method = "mean")
        allImages <- subtractBackground(bg = stillBack)

        #4.2 particle identification
        partIden <- identifyParticles(sbg = allImages,
                                      threshold = 0.08,
                                      pixelRange = c(20,500),
                                      autoThres = FALSE,
                                      select = "light")
        partIdenNN <- update(partIden, finalNN)

        #4.3 tracking
        recordsNN <- trackParticles(partIdenNN,
                                    L = 80, R = 6,
                                    weight = c(0.4, 0.1, 0.3),
                                    costconstant = TRUE,
```

```
                                        logsizes = FALSE)
        #4.4 tracking measurements
        popsize <- summary(recordsNN, incThres = minFrames)$N #population size
        for(track in 1:popsize){
                sizes <- summary(recordsNN, incThres = minFrames)$sizerecord[track,]
                coordinates <- data.frame(frame = seq(1:60),
                        x = summary(recordsNN, incThres = minFrames)$trackrecord[track,1:60,1],
                        y = summary(recordsNN, incThres = minFrames)$trackrecord[track,1:60,2])
                measurements <- c(samples[i], # sample name
                                summary(recordsNN, incThres = minFrames)$particles[track,1], #ID
                                coordinates[!is.na(coordinates$x),]$frame[1], #first frame
                                rev(coordinates[!is.na(coordinates$x),]$frame)[1], #last frame
                                length(na.omit(sizes)), #detections
                                mean(sizes, na.rm = TRUE), #size
                                sqrt(((rev(coordinates[!is.na(coordinates$x),]$x)[1] –
                                                coordinates[!is.na(coordinates$x),]$x[1])^2) +
                                        ((rev(coordinates[!is.na(coordinates$x),]$y)[1] –
                                                coordinates[!is.na(coordinates$x),]$y[1])^2)) #displacement
                                )
                results[nrow(results) + 1,] <- measurements
                }

        # 4.5 generating pdf for review/checks
        pdf(paste0(direc,'.pdf'))
        plot(partIdenNN, frame = 1, incThres = minFrames)
        plot(recordsNN, type='trajectories', incThres = minFrames)
        dev.off()

        # 4.6 cleaning up large objects
        rm("allFullImages", "stillBack", "allImages")
}


## 5. Saving results to csv file
write.csv(results, ‘tracking_results.csv’)
```

UPLC protocol for the quantification of eugenol in Elendt M7

The concentration of eugenol over time was measured in the exposure medium (Elendt M7 medium) with a UPLC-MS system (UPLC: I-class FTN, Waters Acquity, USA and MS: Qtrap 6500, Sciex, Canada) using a C18 column (UPLC BEH C18 [2.1mm×50 mm, 1.7 μm], Waters Acquity, USA). Two ternary mixtures of water, methanol and formic acid were used as eluents (eluent A: 90/10/0.1% v/v and eluent B: 10/90/0.1% v/v respectively). The gradient used for the chromatography can be seen in Table S1. The parameters of the MS are detailed in Table S2 and the settings of the multi-reaction monitoring (MRM) in positive ion mode used as the scan mode in Table S3.

Eugenol standard (purity >98%, Sigma-Aldrich, Germany) was used to prepare a stock solution. A calibration with a range of 0-200 ppb eugenol was made from this stock solution. One mL of each sample from the experiment was transferred into 1.5 mL vials and an internal standard (eugenol-d3, MedChemExpress, USA) was added to reach a concentration of 50 ppb of eugenol-d3 in each sample. All samples were measured twice. The peaks were integrated based on the expected retention time by the Sciex software and the area of the peaks were used to quantify eugenol concentrations.

SUPPORTING TABLES

Table S1. Detailed settings for automated daphnid tracking

**1. Recording phase**	
Cuvette dimensions	20 cm x 15 cm x 3 cm
Camera resolution	1920 x 1080 pixels
Frame rate	30 FPS
Recording days for populations that were exposed to the bentonite nanocarrier, the sepiolite nanocarrier, the bare bentonite nanocarrier combined with pure eugenol, and the bare sepiolite nanocarrier combined with pure eugenol.	Day 0, 1, 2, 4, 7, 8, 9 and 12 of exposure.
Recording days for the populations that were exposed to the bare bentonite nanocarrier, the bare sepiolite nanocarrier, and pure eugenol.	Day 0, 1, 2, 3, 4, 7, 8, 9, 10 and 11 of exposure.
**2. Image pre-processing phase**	
Image-sequence frame rate	2 FPS
Median filter	2 pixel diameter
**3. Trackdem settings for daphnid tracking**	
Still background method	'mean'
partIden size filter	20 to 500 pixels<br>(35 mm to 88 mm)[1]
partIden intensity filter	0.08
Neural network reference movie	Sepiolite nanocarrier, day 9
Neural network training set	64 true positive, 158 false negative
Neural network statistic	recall
Neural network layers	4
Neural network repetitions	20
Neural network training:test:validation split	6:2:2
trackParticles cost for linking particles (L)	80

trackParticles maximum number of linked frames (R)	6
trackParticles weights: spatial coordinates; particle size; predicted vs. observed location	0.4; 0.1; 0.3
recorded tracking parameters	track ID;<br>number of the first and final frame;<br>number of particle detections;<br>mean particle size of detected particles;<br>sum of displacement between consecutive frame
[1] for the first 3 days of exposure to the bare nanocarriers and pure eugenol, the size filter was set to 15 to 500 pixels, and the intensity filter was set to 0.12 due to higher light exposure settings	

Table S2. Gradient used in the UPLC for the quantification of eugenol

Min.	Flow (Ml/Min)	% Eluent A	% Eluent B
0.00	0.50	90	10
0.10	0.50	75	25
0.70	0.50	10	90
1.25	0.50	10	90
4.00	0.50	90	10
4.10	0.50	90	10

Table S3. Source and systems settings of the MS for eugenol quantification

Ion Source	Turbo Spray
Curtain Gas	40
Collision Gas	Medium
Ion Spray Voltage	500
Temperature	550
Ion Source Gas 1	60
Ion Source Gas 2	60
Declustering Potential	25
Entrance Potential	10
Collision Energy	17
Collision Cell Exit Potential	15

Table S4. Settings of the MRM mode for eugenol quantification

Precursor	Product	Time (ms)	ID
165	124	20	Eugenol
165	137	20	Eugenol
168	127	20	Eugenol-d3
168	140	20	Eugenol-d3

Table S5. Comparison of nominal and actual concentrations of eugenol in combined treatments of bare nanocarriers and eugenol in population-level tests. Total nanocarrier weights (nanocarrier + loading) are presented. Eugenol concentrations and release rates correspond to initially released eugenol concentrations. The total area under curves (AUC) was derived from aqueous eugenol concentration measured over the full exposure duration (Fig. 3). Decay curves are presented in Figure S5.

**Treatment**	**Exposure level**	**nominal [nanocarrier] ($mg{\cdot}L^{-1}$)**	**nominal [eugenol] ($mg{\cdot}L^{-1}$)**	**actual [eugenol] ($mg{\cdot}L^{-1}$)**	**AUC ($mg{\cdot}L^{-1}{\cdot}12d$)**
combined eugenol+ bentonite	low<br>medium	11.7<br>23.5	2.9<br>5.9	2.52 ± 0.01<br>4.93 ± 0.04	10.2<br>28.1
combined eugenol+ sepiolite	low<br>medium	24.2<br>48.3	1.8<br>3.6	1.49 ± 0.03<br>3.24 ± 0.24	6.1<br>16.0

SUPPORTING FIGURES

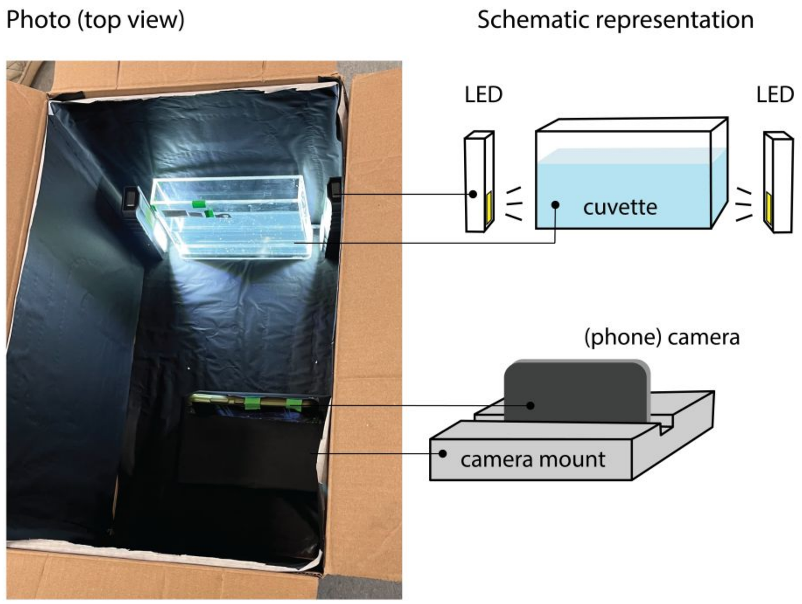

Figure S1. Video recording setup used to acquire automated daphnid tracking footage.

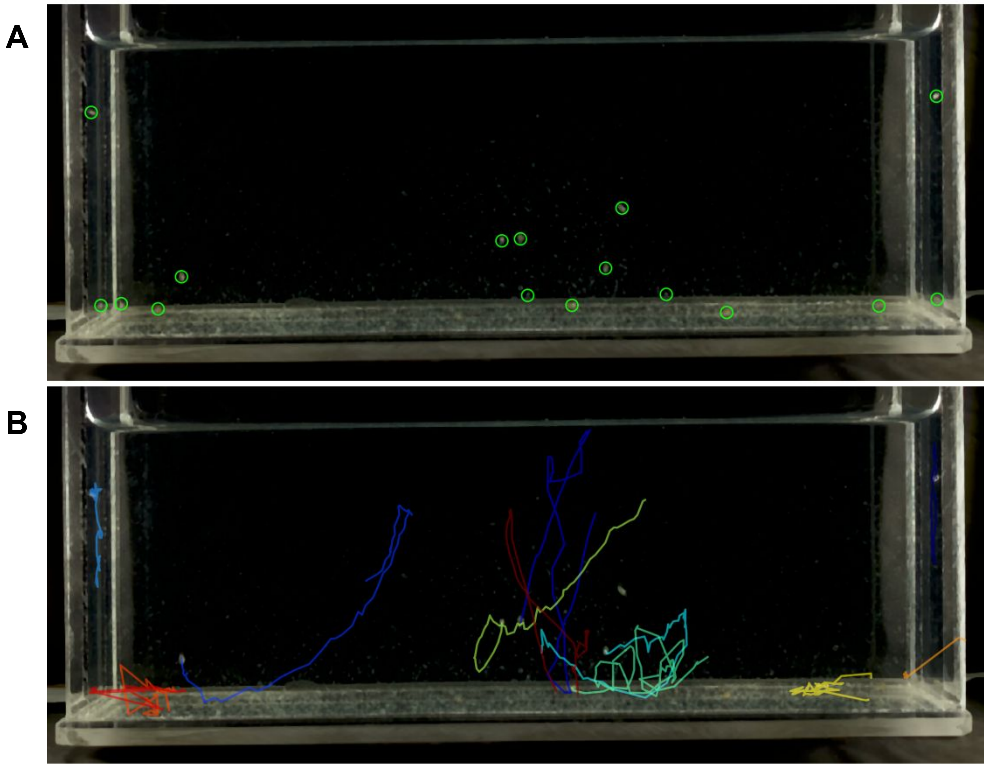

Figure S2. Examples plots from Trackdem of the detected particles (A) and recorded particle trajectories (B).

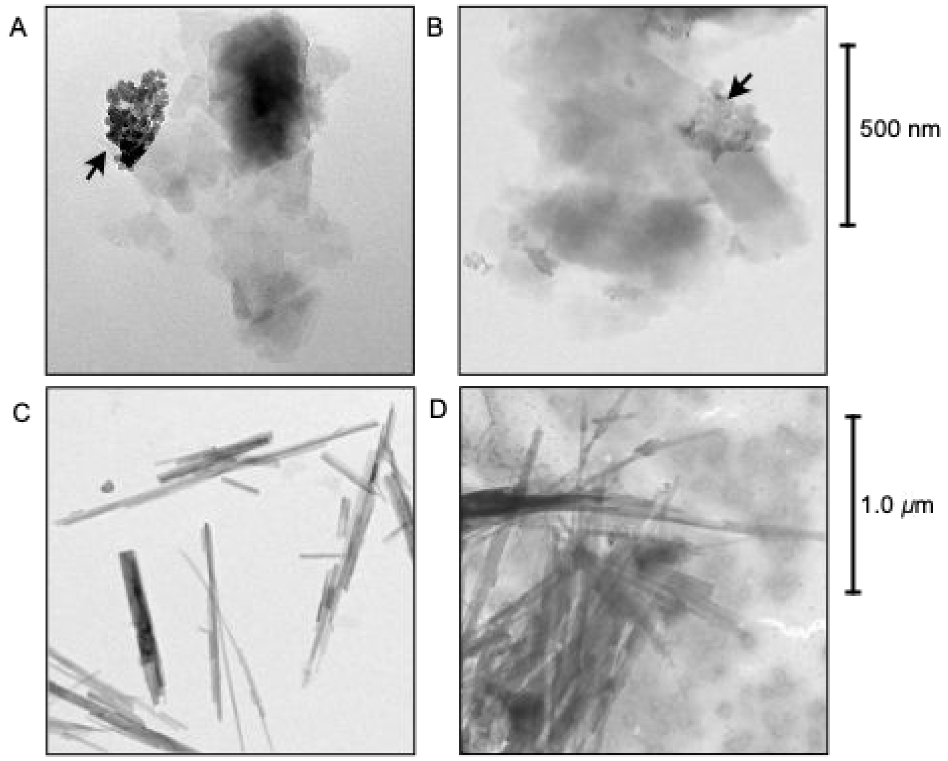

Figure S3. Transmission electron microscope micrographs of bare bentonite particles (A), the bentonite nanocarrier (B), bare sepiolite particles (C) and sepiolite nanocarrier (D). The 500 nm-scalebar applies to panel A and B, and the 1 μm-scale bar applies to panel C and D). Arrows indicate contamination.

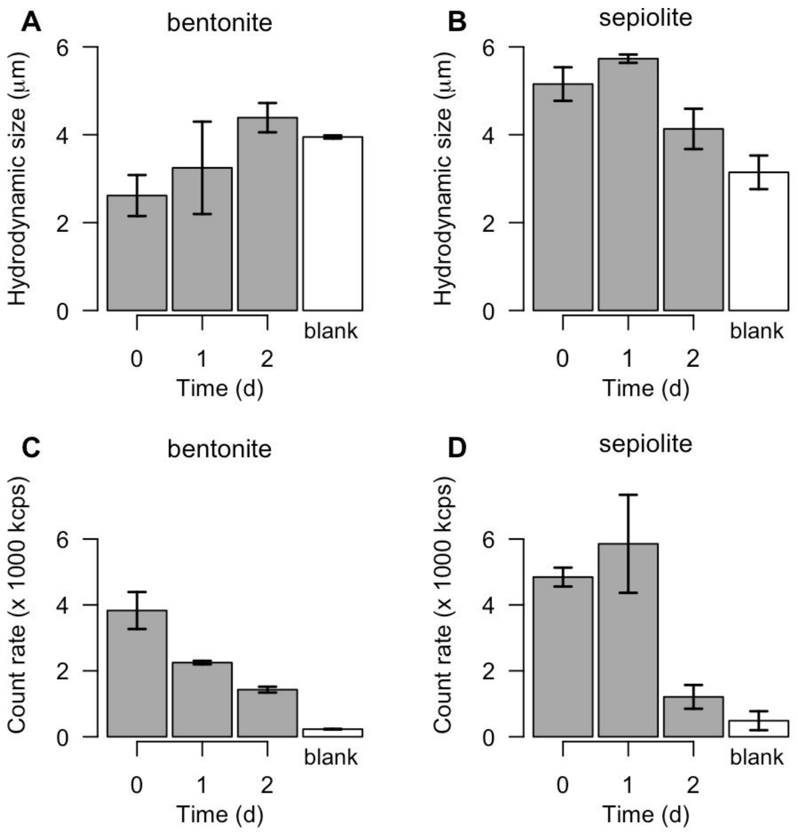

Figure S4. Dynamic Light Scattering measurements over time of z-average hydrodynamic size (A,B) and derived count rate (C,D) of bentonite (A,C) and sepiolite (B,D) nanocarriers.

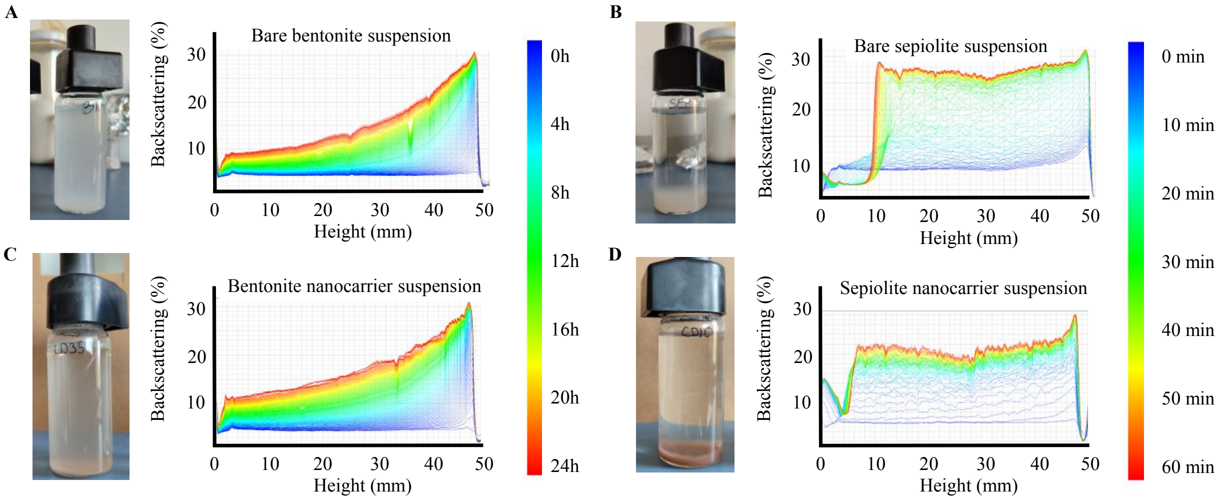

Figure S5. Suspensions stability by studying changes in backscattering over time using Turbiscan equipment (time scale in colored bar scale at the right of the graph): A) bare bentonite suspension; B) bare sepiolite suspension; C) bentonite nanocarrier suspension; and D) sepiolite nanocarrier suspension. The 0 mm height corresponds to the bottom of the test flask, and the 50 mm to its top. The photographic images in each graph correspond to the end of the suspension stability test.

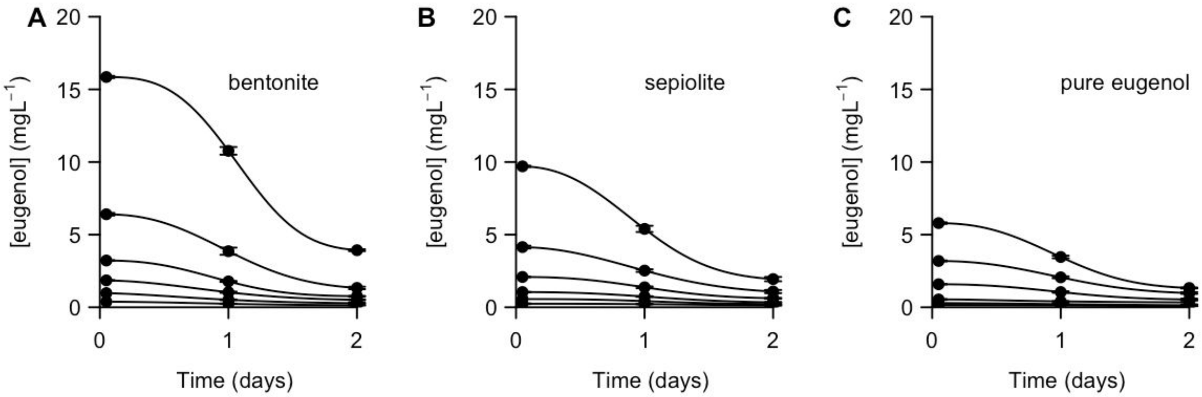

Figure S6. Actual concentrations of eugenol measured in the exposure medium of acute immobilization tests performed for loaded bentonite (A), loaded sepiolite (B) and pure eugenol (C). From high to low actual concentrations, the curves correspond to nominal exposure concentrations of 500, 200, 100, 50, 25 and 10 mg nanocarrier L^{-1} (A and B) or 5, 2, 1, 0.5, 0.25 and 0.1 mg eugenol L^{-1} (C). Decay curves were fitted to the measurements using the four parameter Weibull model.

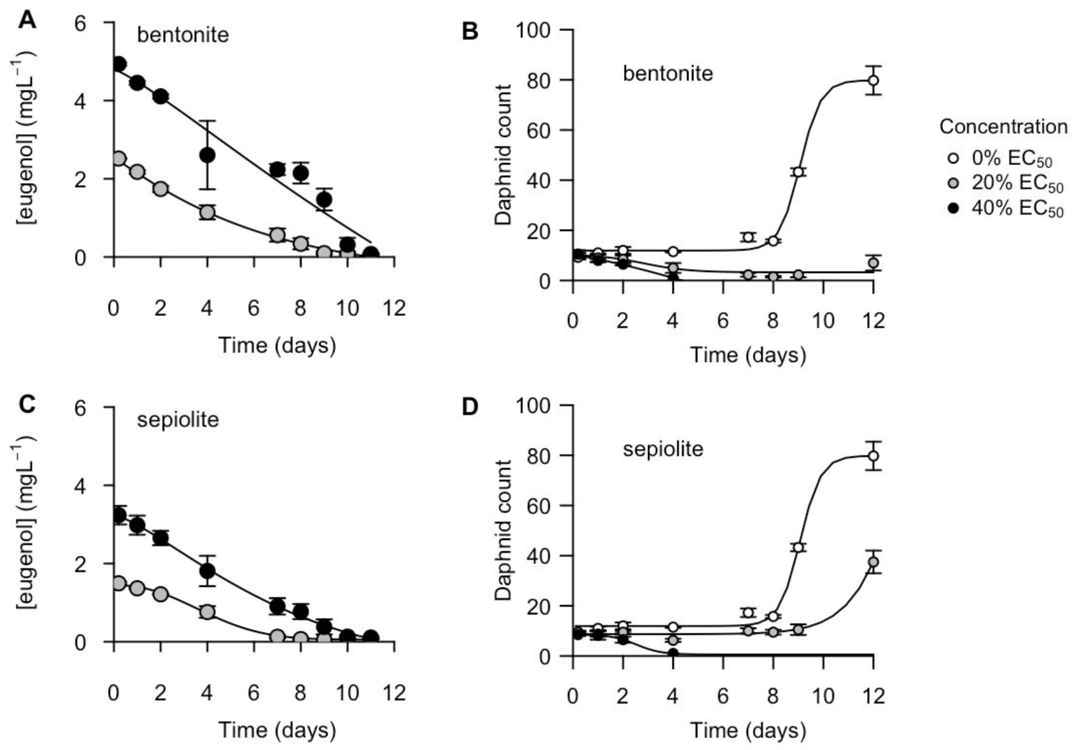

Figure S7. Aqueous concentrations of eugenol (A,C) and population size (B,D) in combined exposure treatments to bare nanocarriers and eugenol in population-level tests. Panel A and B present results for the bare bentonite nanocarrier, and panel C and D present results for the bare sepiolite nanocarrier.

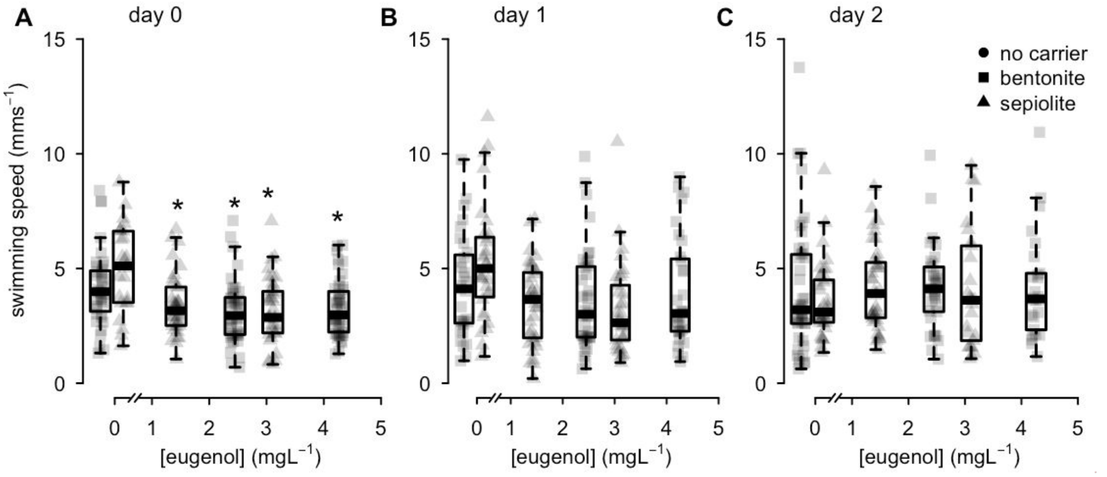

Figure S8. Swimming speed of *D. magna* individuals following 1 day (A,B) or 2 days (C,D) of exposure to the bare bentonite nanocarrier (squares), bare sepiolite nanocarrier (triangles) or pure eugenol (circles) as a function of actual concentrations of released eugenol. Significant

relationships between the released eugenol concentrations and swimming speed ($p < 0.05$) are indicated with an asterisk above the boxplots of the bare nanocarriers.
